# Deep learning-based automated pipeline for blood vessel detection and distribution analysis in multiplexed prostate cancer images

**DOI:** 10.3389/fbinf.2023.1296667

**Published:** 2024-01-23

**Authors:** Grigorios M. Karageorgos, Sanghee Cho, Elizabeth McDonough, Chrystal Chadwick, Soumya Ghose, Jonathan Owens, Kyeong Joo Jung, Raghu Machiraju, Robert West, James D. Brooks, Parag Mallick, Fiona Ginty

**Affiliations:** ^1^ GE Research, Niskayuna, NY, United States; ^2^ Department of Computer Science and Engineering, The Ohio State University, Columbus, OH, United States; ^3^ Department of Pathology, Stanford University School of Medicine, Stanford, CA, United States; ^4^ Department of Urology, Stanford University School of Medicine, Stanford, CA, United States; ^5^ Canary Center for Cancer Early Detection, Department of Radiology, Stanford University School of Medicine, Stanford, CA, United States

**Keywords:** deep learning, blood vessel detection, pathology image analysis, prostate cancer, automated segmentation

## Abstract

**Introduction:** Prostate cancer is a highly heterogeneous disease, presenting varying levels of aggressiveness and response to treatment. Angiogenesis is one of the hallmarks of cancer, providing oxygen and nutrient supply to tumors. Micro vessel density has previously been correlated with higher Gleason score and poor prognosis. Manual segmentation of blood vessels (BVs) In microscopy images is challenging, time consuming and may be prone to inter-rater variabilities. In this study, an automated pipeline is presented for BV detection and distribution analysis in multiplexed prostate cancer images.

**Methods:** A deep learning model was trained to segment BVs by combining CD31, CD34 and collagen IV images. In addition, the trained model was used to analyze the size and distribution patterns of BVs in relation to disease progression in a cohort of prostate cancer patients (*N* = 215).

**Results:** The model was capable of accurately detecting and segmenting BVs, as compared to ground truth annotations provided by two reviewers. The precision (P), recall (R) and dice similarity coefficient (DSC) were equal to 0.93 (SD 0.04), 0.97 (SD 0.02) and 0.71 (SD 0.07) with respect to reviewer 1, and 0.95 (SD 0.05), 0.94 (SD 0.07) and 0.70 (SD 0.08) with respect to reviewer 2, respectively. BV count was significantly associated with 5-year recurrence (adjusted *p* = 0.0042), while both count and area of blood vessel were significantly associated with Gleason grade (adjusted *p* = 0.032 and 0.003 respectively).

**Discussion:** The proposed methodology is anticipated to streamline and standardize BV analysis, offering additional insights into the biology of prostate cancer, with broad applicability to other cancers.

## 1 Introduction

Prostate cancer is a heterogeneous multi-focal disease, presenting variations in underlying genetic mutations and biology, tumor size and growth patterns (Tolkach and Kristiansen, 2018), (Boyd et al., 2012). Such variations result in differences in aggressiveness and response to therapy (Wallace et al., 2014). Comprehending the underlying biology and progression mechanisms of prostate cancer is important for determining diagnostic and personalized treatment strategies (Wallace et al., 2014).

Digital pathology image analysis algorithms have shown great promise in elucidating the pathophysiology of prostate cancer (Goldenberg et al., 2019). Advances in machine learning and computer vision have significantly enhanced the capability of these algorithms to perform detection, segmentation, labelling and classification of multiple histological features associated with the molecular and spatial characteristics of diseases (Gupta et al., 2019). For example, analysis of hematoxylin and eosin (H&E) images, including analysis of color, texture and morphological features (Tabesh et al., 2007), does not require any additional molecular or biomarker stains. Various conventional machine learning (ML) techniques, including support vector machine (SVM), k nearest-neighbor (kNN) and Gaussian classifiers have been employed to automatically distinguish between H&E images with and without tumor, as well as perform Gleason grading, which is a well-established methodology for classifying prostate cancer stages (Bostwick, 1994). More recently, deep learning approaches (DL) have been proposed for Gleason grading of prostate biopsy images and demonstrated accurate staging compared with pathologists’ assessment, and good generalization capabilities in large datasets obtained from different institutions (Bulten et al., 2020; Nagpal et al., 2020; Kott et al., 2021).

Angiogenesis plays a key role in cancer progression (van Moorselaar and Voest, 2002; Mucci et al., 2009; Li and Cozzi, 2010). The formation of blood vessels (BV) facilitates delivery of nutrients and oxygen that are vital for the development of solid tumors, while the use of antiangiogenic treatment has shown potential to impede tumor advancement, albeit with varying levels of success (Folkman, 2006), (Ioannidou et al., 2021). Moreover, angiogenesis can promote the spread of cancer cells to other parts of the body through the vasculature, resulting in metastasis (Fukumura and Jain, 2007). Therefore, gaining a deeper comprehension of the vasculature and surrounding biology holds the potential to yield improved prognostic indicators and treatment strategies. Manual identification of BV in H&E images is challenging, time consuming and may suffer from inter-rater variabilities. To address those challenges, DL-based approaches for BV detection and classification in H&E histopathology images of lung adenocarcinoma (Vu et al., 2019) (Yi et al., 2018), oral cancer (Fraz et al., 2018), gastric cancer (Noh et al., 2023), pancreatic cancer (Kiemen et al., 2022) and kidney tissue (Bevilacqua et al., 2017), have been previously proposed. While many studies have focused on analysis of blood vessels in H&E images, immunostaining of CD31 (endothelial cell protein) and CD34 (vascular endothelial cell protein and neo angiogenesis) provide higher specificity for BV identification (Miyata et al., 2015). In addition, collagen IV immunostaining which is present in vascular basement membranes can provide complementary information on identification of BV (Gross et al., 2021). Recent advancements in multiplexed imaging allow staining of multiple biomarkers in a single sample, allowing integration of colocalized and co-expressed proteins and spatial analysis of surrounding biology.

In this study an automated pipeline for BV detection and distribution analysis in pathology images for prostate cancer staging is presented using a combination of CD31, CD34 and collagen IV. A deep-learning model was trained with a limited dataset consisting of 29 patient core images, and BV were segmented by combining 1) all three markers; 2) CD31 and CD34; 3) CD31 alone. The most robust performance was found using all three markers, while the highest false positive rate was obtained by using CD31 alone. The three-marker model was used to analyze the size and distribution patterns of BVs in relation to disease progression in a cohort of prostate cancer patients (N = 215). Blood vessels count and size were significantly associated with Gleason grade, while patients with highest BV counts had significantly higher risk of recurrence.

## 2 Materials and methods

### 2.1 Patient cohort

This study was performed in accordance with ethical guidelines for clinical research with the approval of the Institutional Review Board of Stanford University (IRB: 11612). All patients included in this study (N = 215) were aged between 45 and 78 years (average 64.5 years) and underwent surgery for histologically proven prostate cancer between 1985 and 1997. Patient’s exclusion criteria were as follows: postoperative mortality within 30 days; a limited follow-up period of less than 3 years in cases without recurrence; synchronous multiple cancers; No patient received preoperative chemotherapy or radiotherapy. Average follow-up was 95.5 (standard deviation (SD) 50.9) months and 5-year recurrence rate of 21%. Three tissue microarrays (TMAs) were constructed with up to four 0.6-mm-diameter pathologist selected cancer regions and reference/fiducial cores. The dataset consisted of a total 849 cores from patients with prostate cancer diagnosis (from 226 patients, and up to four cores per patient). After excluding cases with missing outcome data, image artefacts, we analyzed 749 cores from 215 patients. Summary statistics for clinical and demographic data are shown in Table 1.

**TABLE 1:** Summary of patient demographics.

	Biochemical Recurrence (N = 55)	No Recurrence (N = 160)	Total (N = 215)
**Ethnicity**			
African American	1 (1.8%)	0 (0.0%)	1 (0.5%)
Asian	4 (7.3%)	2 (1.2%)	6 (2.8%)
Caucasian	47 (85.5%)	133 (83.1%)	180 (83.7%)
Unknown	3 (5.5%)	25 (15.6%)	28 (13.0%)
**Age**			
Mean (SD)	63.946 (6.358)	64.541 (6.517)	64.389 (6.467)
Range	43.397–76.721	45.195–78.671	43.397–78.671
**T stage**			
T2	15 (27.3%)	124 (77.5%)	139 (64.7%)
T3	37 (67.3%)	36 (22.5%)	73 (34.0%)
T4	3 (5.5%)	0 (0.0%)	3 (1.4%)
**N stage**			
N-Miss	1	6	7
N0	46 (85.2%)	153 (99.4%)	199 (95.7%)
N1	8 (14.8%)	1 (0.6%)	9 (4.3%)
**Grade.group**			
1	1 (1.8%)	39 (24.4%)	40 (18.6%)
2	29 (52.7%)	96 (60.0%)	125 (58.1%)
3	25 (45.5%)	24 ((15.0%)	49 (22.8%)
4	0 (0.0%)	1 (0.6%)	1 (0.5%)
**Follow up Time (Month)**			
Mean (SD)	42.810 (48.298)	113.591 (50.847)	95.484 (58.888)
Range	0.500–223.733	5.000–291.800	0.500–291.800

The bold values indicate the type of data in the respective cells of the table.

### 2.2 Multiplexed immunofluorescence imaging of TMAs

TMAs underwent multiplexed immunofluorescence (MxIF) imaging on a calibrated Cell DIVE imager (Leica Microsystems, Issaquah, WA, United States of America) using a 20 × 0.75 NA objective, with a resulting pixel size of 0.325 µm/pixel and 16-bit images (no pixel binning). Automated software enabled system calibrations using a multi-function calibration plate and automated image processing allowed for field flattening, autofluorescence (AF) removal, and image registration. The Cell DIVE imager has an SSI light engine with five independently controlled light sources. Light is delivered to the sample through a 1.5 mm fiber optic cable that provides uniform illumination across the specimen. Exposure times for each channel were determined for each biomarker based on a visual assessment of fluorescent intensity and set to achieve ∼75% of the dynamic range of the camera without saturating any of the pixels; exposure times ranged as follows: DAPI, 20 m; Cy2, 100 m; Cy3, 20–1000 m; Cy5, 15–1500 m; and Cy7, 400–1500 m. Focus is determined using a hardware laser autofocuser that is part of microscope (Gerdes et al., 2013).

A total of 50 proteins were imaged, and the analysis for this paper focused on four of those including CD31, CD34, collagen IV, and nuclear marker DAPI. All antibodies were characterized using a previously published protocol (McDonough et al., 2020). In brief, TMA slides were de-paraffinized and rehydrated, underwent a two-step antigen retrieval, and were blocked with serum overnight. Prior to antibody staining, tissue was DAPI stained and image in all channels of interest to collect background autofluorescence. Following this, tissue was stained with up to three Antibodies per cycle for 1 h at room temperature using a Leica BOND-MAX autostainer and reimaged to capture antigen-specific signal. After imaging of the stained sample, tissues underwent a dye inactivation step to remove the dye signal and were re-imaged to measure background fluorescence intensity. Images were processed for illumination correction, registered across all rounds using DAPI, and autofluorescence subtracted. These cycles were repeated until all targets of interest were imaged.

### 2.3 Data pre-processing for blood vessel segmentation

Samples with low, medium, and high average staining intensity of CD31 and CD34 were randomly selected across the three TMAs ((N = 30; 10 cores per TMA). The selected cores were annotated by either one, or two expert biologist, using the manual annotation tools provided by open source software QuPath (Bankhead et al., 2017), and a script was applied to export annotation coordinates for the deep learning algorithm. Manual annotation was carried out based on the CD31 images, by also cross-checking other available markers, including CD34, collagen IV, and nuclear marker DAPI. One core was excluded from analysis due to insufficient quality of BV annotations, leading to twenty-nine cores with a total of 1,327 annotated BVs.

**FIGURE 1:**
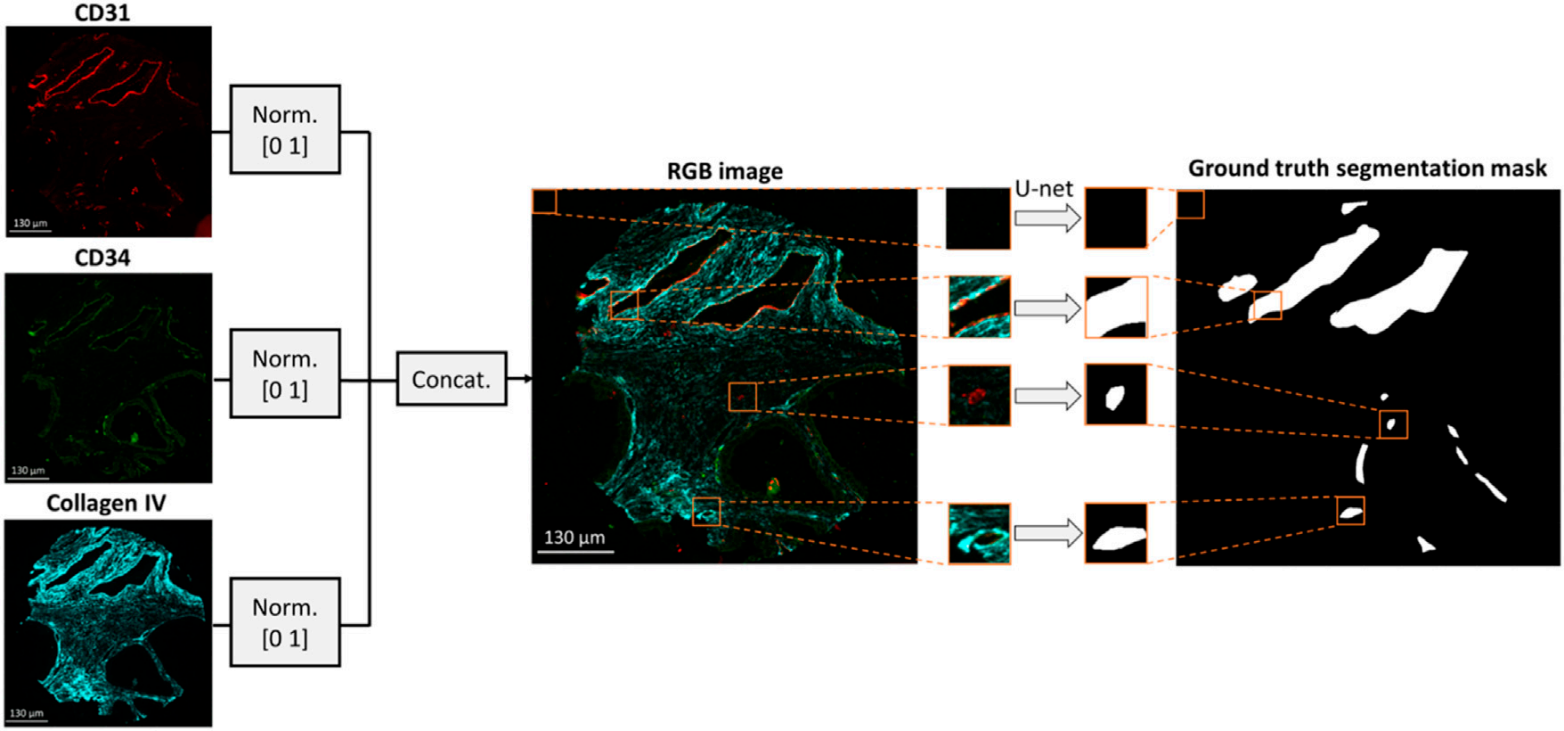
Dataset formation for BV segmentation. An RGB image is formed by concatenating the CD31, CD34 and collagen IV multiplexed images. The resulting RGB image is divided into 128 × 128 × three patches which are fed into a U-net to generate the respective patches of the segmentation mask.

The data pre-processing methodology is depicted in Figure 1. Images of CD31, CD34 and collagen IV were scaled within a range of [0 1] based on their minimum and maximum intensities and concatenated to form a 3-channel RGB image. Concatenation was performed to combine BV information from multiple stains, thus reducing the impact of random variation or outliers that may be present in a single image. Independent annotated patient core images (N = 23) were selected to form the training/validation dataset, while the remaining images (N = 6) were held out to test the generalization capability of the model. The six cores in the test dataset included two cores from each of the three TMAs. To enhance the robustness of the model performance assessment, the six cores in the test dataset were also annotated by a separate expert biologist, obtaining thus two separate ground truth segmentation masks. The inter-observer variability between the two annotators was calculated in terms of average number of annotated vessels per core. Each training/validation image was divided to 128 × 128 × 3 patches with no overlap. Due to the overrepresentation of pixels with negative values in the training dataset (i.e., regions negative for CD31 staining), 80% of negative image patches were discarded, while positive patches were augmented by a factor of four by performing 90 ° patch rotations. The augmentation process resulted to a total of 24,744 patches, which were divided into training and validation datasets with a split of 22,270/2,474. The same division and augmentation strategy was also performed on the ground truth binary segmentation masks to maintain the information on the position of vessels.

To determine whether including all three markers (CD31, CD34 and collagen IV) improves the reliability of the BV segmentation method, two additional datasets were formed with either 2-channel patches of size 128 × 128 × 2 resulting from concatenation of CD31 and CD34 images, or grayscale patches of size 128 × 128 × 1 including only the CD31 images. The control dataset was derived from the same images and using the same augmentation strategy, as the RGB patches.

### 2.4 Deep learning model architecture

**FIGURE 2:**
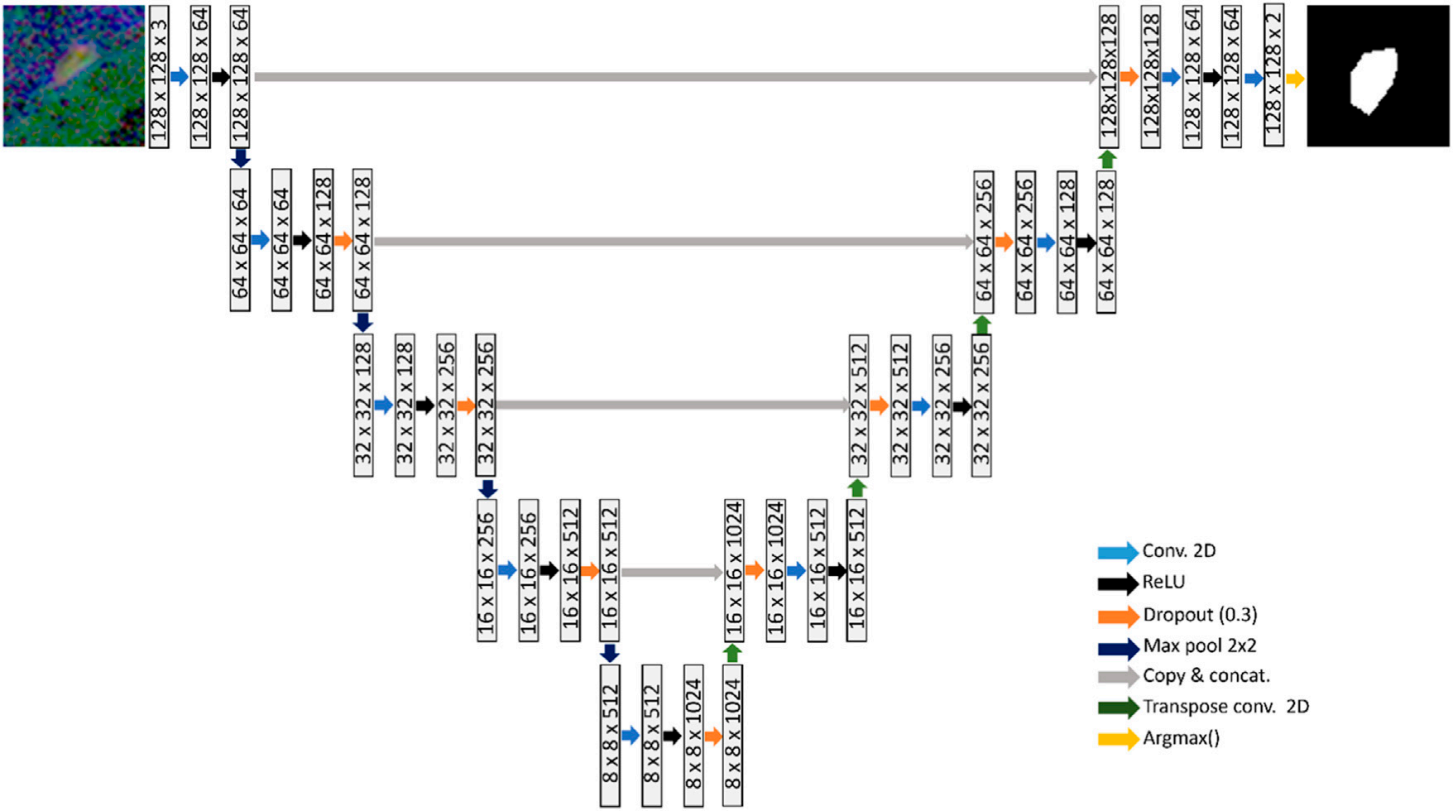
U-net consisting of four down-sampling and four up-sampling layers, which is trained to learn the BV segmentation task.

A U-net model was used to learn the segmentation task, as shown in Figure 2 (Ronneberger et al., 2015). The U-net architecture comprises of an encoder network that captures increasingly higher-level features from an input image. The encoder is followed by a decoder that up-samples these features to reconstruct a segmentation map. Each layer consists of 3 × 3 convolutions, followed by rectified linear unit activation and dropout regularization with a dropout rate of 0.3. Down-sampling is carried out via 2 × 2 max-pooling operator. To gradually increase the spatial dimensions and reduce the number of channels of the feature maps to the original size, transpose convolutions are used in the decoding stage.

Skip connections are employed at various scales that concatenate the encoder output at each down-sampling layer to the respective up-sampling layer in the decoder. The purpose of the skip-connections is to propagate features from the same scale to each decoding layer, facilitating learning of global and local contextual information. A softmax function is applied at the final up-sampling stage of the U-net, to convert the output into probability scores that indicate the likelihood each pixel belonging to each class (Negative class: No presence of blood vessels; Positive class: Presence of blood vessels). The argmax operator is finally applied to assign each pixel to a class based on the probability scores.

### 2.5 Model training and testing

Three models were trained by using either the 3-channel RGB (CD31, CD34 and collagen IV), or the 2-channel comprising CD31 and CD34, or the grayscale image patches including just the CD31 staining. Each model was trained using the Adam optimizer, with a batch size of 32, learning rate of 0.0001, and the sparse categorical cross entropy (CCE) as loss function. Early stopping was used with a patience of seven epochs, and a maximum of 100 epochs. The pixel classification accuracy, defined as (TP + TN)/(TP + TN + FP + FN) was monitored during the training process, both in the training and validation datasets. The weights of the model with highest validation accuracy were saved and used to infer the model in the test dataset. The model architecture and training was implemented in the deep learning library Keras/Tensorflow (Oreilly, 2023), (Abadi et al., 2016). The training process was carried out on an NVIDIA Tesla V100 SXM2 (NVIDIA, Santa Clara, CA, United States of America) graphics processing unit (GPU).

Model inference was carried out on each core image in the test dataset, by dividing them in 128 × 128 patches with a 75% horizontal and vertical overlap. The output probability resulting by each patch was converted to a binary mask patch by setting each pixel to either 0 or 1 based on the argmax of the respective probability scores. The resulting binary mask patches were stitched together to reconstruct the total segmentation mask, by averaging in the overlapping regions. To make the segmentation reconstruction more robust to false positives, all pixels with an average value <1 were set to 0. In other words, a pixel is assigned to the positive class, only if it is consistently classified as 1 across all patches. To remove noisy artifacts and distortions resulting from patch stitching, the reconstructed mask was subjected to morphological opening. Finally, morphological closing was carried out to fill holes in the vessel segmentation mask. A disk with radius of three pixels was used as morphological structuring element. The total time taken for the model to reconstruct the total segmentation mask for a single core was equal to 108.9 s.

### 2.6 Model accuracy assessment

Using the manually annotated images, the pixel-wise agreement between the ground truth (M_GT_) and generated (M_Gen_) segmentation masks was evaluated in the test dataset against each of the two ground truth annotations, in terms of dice similarity coefficient (DSC), as follows:

$$\text{DSC}=\frac{2*\left|M_{\text{GT}}\cap M_{\text{Gen}}\right|}{\left|M_{\text{GT}}\right|+\left|M_{\text{Gen}}\right|}$$ (1)

In addition, the vessel detection capability of the model was assessed by calculating the precision (P) and recall (R):

$$P=\frac{\text{TP}}{\text{TP}+\text{FP}},R=\frac{\text{TP}}{\text{TP}+\text{FN}}$$ (2)

Where TP, FP and FN stand for True Positives, False Positives and False Negatives, respectively. Vessels in M_Gen_ presenting overlap with vessels in M_GT_ were classified as TP, while those without overlap were marked as FP. Vessels present in M_GT_ that were not identified in M_Gen_ were considered considered as FN.

### 2.7 Blood vessel size and distribution analysis

Blood vessels were automatically segmented using the best performing model in all 749 cores that met the inclusion criteria, resulting to a total of 40749 segmented objects were segmented from 758 cores. Vessel size (number of pixels) of each segmented area were extracted for each blood vessel. To remove any image artefact, For blood vessels larger than 6000 pixels, visual inspection was performed to filter out potential artifacts which corresponds to 625um^2^ and 4% of all segmented objects. This includes 1656 objects involving 621 cores—ultimately reviewing 80% of the samples. From this process, 139 segmented objects were filtered out from the analysis out of 1656. Subsequently, standard summary statistics and histograms were generated for vessel size as well as core-level vessel count/total area. To evaluate the associations with Gleason grade and patient outcomes, core-level data on the vessel counts and total area was aggregated and averaged and divided into tertiles (low/medium/high vessel count groups). ANOVA analysis was applied to compare differences in vessel counts and total area between the Gleason grade groups. *p*-values were adjusted using the Benjamin-Hochberg method for multiple hypothesis tests. Kaplan-Meier plots were generated to evaluate the recurrence rate differences among these three groups.

## 3 Results

### 3.1 Vessel segmentation performance

**FIGURE 3:**
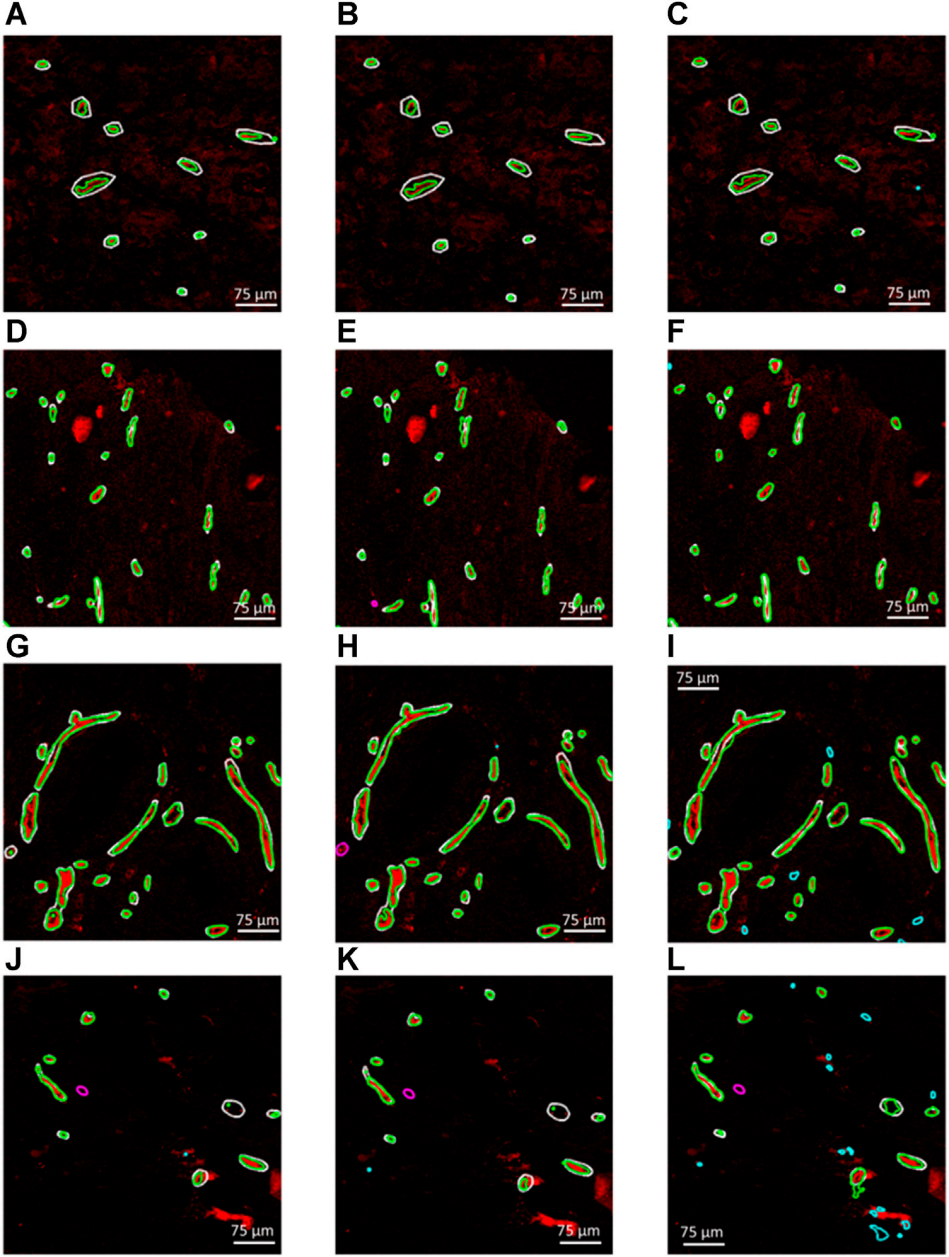
**(A–C)**, **(D–F)**, **(G–I)** and **(J–L)** Four example CD31 images corresponding to different subjects in the test dataset. The ground truth annotations are overlaid in white outlines. The green, cyan and magenta outlines illustrate the true positive, false positive and false negative predictions that were generated by each trained segmentation model. **(A,D,G and J)** Generated segmentation masks, provided by the RGB (CD31, CD34, collagen IV). **(B,E,H and K)** Generated segmentation masks, provided the 2-channel (CD31 and CD34) model. **(C,F,I,L)** Generated segmentation masks, provided by the grayscale (CD31) model.

Figures 3A–L demonstrate four example CD31 images corresponding to different subjects in the test dataset. The ground truth annotations are overlaid in white outlines. The green, cyan and magenta outlines illustrate the true positive, false positive and false negative predictions that were generated by each trained segmentation model. Figures 3A–L, illustrate the generated segmentation masks, provided by the RGB (CD31, CD34, collagen IV), 2-channel (CD31 and CD34) and grayscale (CD31) models, respectively. Good agreement was found between ground truth and generated segmentation masks, particularly in the case of the RGB model. In the case of the 2-channel and grayscale models, a higher number of FP and FN predictions is present.

The maximum training and validation accuracies observed during the training process were equal to 97.80 and 96.84, respectively, in the case of the RGB model, 97.84 and 96.05, respectively, in the case of the 2-channel model and 97.66 and 95.54, respectively, in the case of the grayscale model. Table 2 summarizes the performance metrics for each of the six patient image cores in the test dataset by using the models trained on RGB, 2-channel and grayscale patches, respectively, with respect to each of the two annotators. The DSC was on average higher in the case of the RGB (Annotator 1: 0.71 (SD 0.07); Annotator 2: 0.70 (SD 0.08)), as compared to the 2-channel (Annotator 1: 0.67 (SD 0.11); Annotator 2: 0.68 SD (0.07)) and grayscale (Annotator 1: 0.67 (SD 0.08); Annotator 2: 0.70 (SD 0.05)) models, indicating improved pixel-wise similarity between M_GT_ and M_Gen_. In addition, the RGB model marked increased precision and recall (Annotator 1: 0.93 (SD 0.04) and 0.97 (SD 0.02); Annotator 2: 0.95 (SD 0.05) and 0.94 (SD 0.07)), compared to the 2-channel (Annotator 1: 0.91 (SD 0.08) and 0.94 (SD 0.05); Annotator 2: 0.92 (0.07 SD) and 0.90 (SD 0.07)) and grayscale (Annotator 1: 0.81 (SD 0.09) and 0.97 (SD 0.02); Annotator 2: 0.82 (SD 0.12) and 0.97 (SD 0.02)) models, demonstrating superior blood vessel detection capability when combining information from CD31, CD34 and collagen IV. The average vessel counts per core, provided by Annotator 1 and Annotator two was equal to 42.67 and 38.33, respectively. The precision was particularly low in the case of the grayscale model (CD31 alone), indicating that a single marker renders the model prone to false positive predictions.

**TABLE 2:** Performance metrics summary for the RGB, 2-channel and grayscale models.

		Annotator 1						Annotator 2					
		S1	S2	S3	S4	S5	S6	S1	S2	S3	S4	S5	S6
**RGB**	**DSC**	0.70	0.68	0.79	0.61	0.68	0.80	0.72	0.61	0.79	0.74	0.60	0.75
	**Precision**	0.95	0.91	0.98	0.91	0.86	0.98	0.95	0.91	0.97	1.00	1.00	0.88
	**Recall**	0.98	0.94	0.98	0.97	1.00	0.98	0.96	0.83	1.00	0.97	0.88	1.00
	**FP**	3	3	1	3	3	1	3	3	2	0	0	5
	**FN**	1	2	1	1	0	1	2	6	0	1	3	0
**2-channel**	**DSC**	0.66	0.63	0.77	0.47	0.70	0.78	0.68	0.57	0.78	0.72	0.62	0.72
	**Precision**	0.93	0.76	0.97	0.91	0.90	1.00	0.90	0.79	0.97	1.00	0.95	0.90
	**Recall**	0.96	0.87	0.92	0.94	1.00	0.98	0.91	0.79	0.93	0.91	0.83	1.00
	**FP**	4	8	2	3	2	0	6	7	2	0	1	4
	**FN**	2	4	5	2	0	1	5	7	4	3	4	0
**grayscale**	**DSC**	0.70	0.67	0.82	0.55	0.73	0.67	0.76	0.62	0.72	0.75	0.70	0.65
	**Precision**	0.90	0.73	0.76	0.82	0.71	0.93	0.90	0.59	0.75	0.92	0.90	0.84
	**Recall**	0.98	0.93	0.98	0.97	1.00	0.98	0.96	0.94	0.98	1.00	0.95	1.00
	**FP**	6	23	18	7	6	3	6	20	19	3	2	7
	**FN**	1	2	1	1	0	1	2	2	1	0	1	0

### 3.2 Vessel size and distribution analysis

**FIGURE 4:**
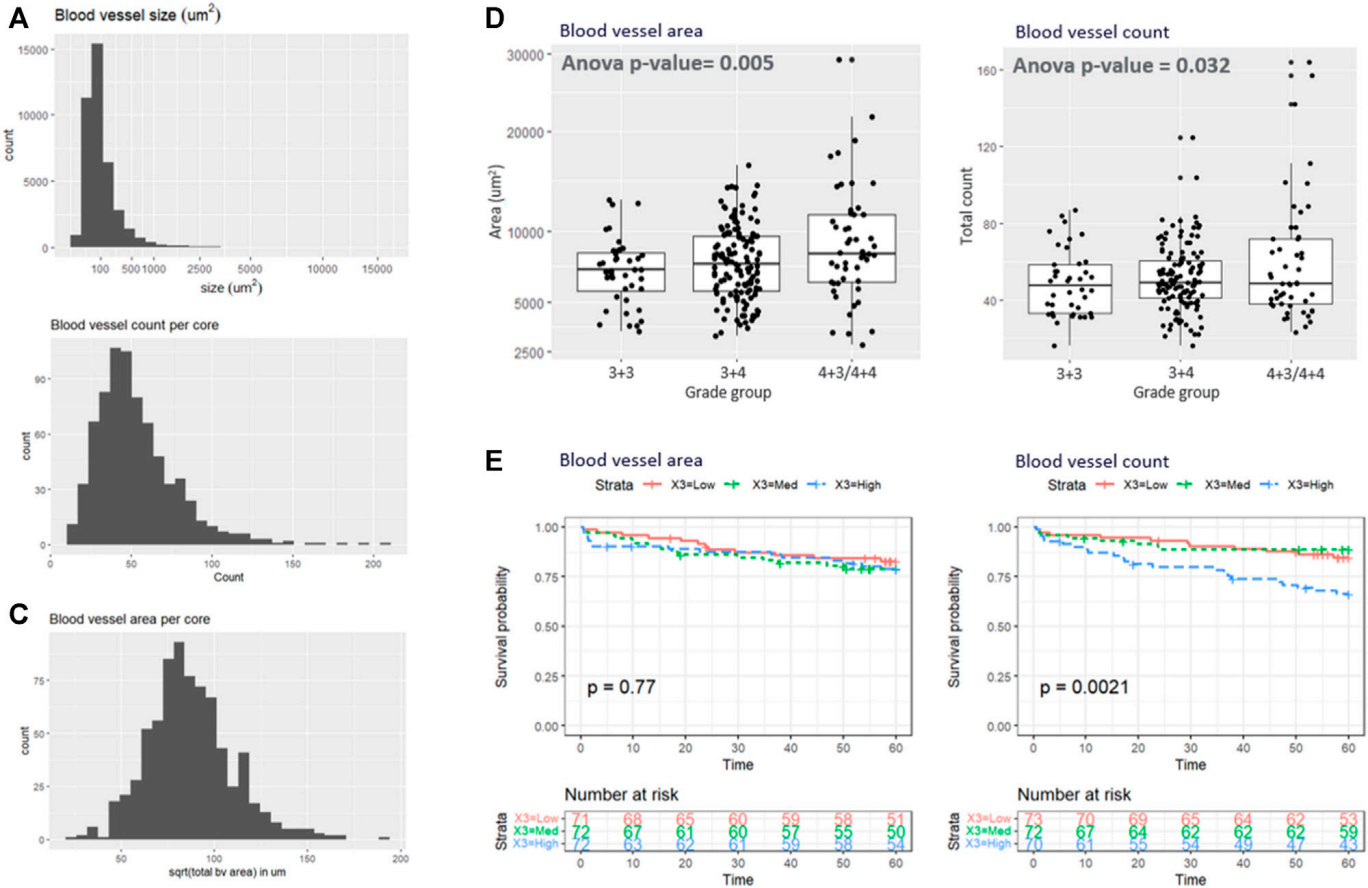
**(A–C)** illustrate the histogram of the **(A)** blood vessel size, **(B)** blood vessel counts per core, and **(C)** total blood vessel area per core. **(D)** Box plot representations of the BV area and total count with respect to the grade group. **(E)** Kaplan-Meyer analysis.

Based on the superior performance of the RGB model in the test set, it was applied to the remaining samples. Figures 4A–C shows the histogram of the blood vessel size, blood vessel counts per core, and total blood vessel area per core. The average segmented blood vessel size was 102 μm^2^. On average, there were 53 blood vessels per core, with a total area of 0.076 mm^2^. Figure 4D shows the box plot representations of the BV area and total count with respect to the Gleason grade group. In average, patients with Gleason grade 3 + 3 had 49 blood vessels (SD = 17) with size of 7111 um2 (SD = 2345) while Patients with Gleason grade 3 + 4 had 51 blood vessels (SD = 17) with size of 7795 um2 (SD = 2762) and patients with Gleason grade 4 + 3 and 4 + 4 had 60 blood vessels (sd = 32) with size of 9514um2 (SD = 5014). ANOVA analysis showed that both the blood vessel count and area is significantly associated with grade group (adj.*p* = 0.032 and 0.005 respectively as depicted in Figure 4D). Figure 4E demonstrates the Kaplan-Meier analysis on the association of the blood vessel counts and size to patient outcome, biochemical recurrence. A significantly higher risk of biochemical recurrence is indicated as the count of blood vessels increases (adj.*p* = 0.0042), while there was no significant association between the outcome and the blood vessel area.

## 4 Discussion

In this study, an automated pipeline was developed for blood vessel detection and distribution analysis using multiplexed images of CD31, CD34 and collagen IV. These markers were selected based on specificity for endothelial cells (CD31 and CD34) and blood vessel basement membrane (collagen IV). While CD31 has been historically used for blood vessel analysis, our hypothesis was that combining three markers expressed or colocalized with blood vessels, would achieve a higher level of segmentation accuracy. A U-net was trained by using different combinations of the three markers, demonstrating that the use of three markers had higher accuracy than CD31 and CD34, or CD31 alone, which was prone to a higher false positive rate. In the absence of all three markers, the proposed pipeline can still use combination of two markers, or just CD31, which still provided acceptable blood vessel detection capability. Using the three-marker combination, we found significant differences between blood vessel counts by Gleason grade and patients with the highest counts had significantly higher risk of biochemical failure within 5 years. This provides a more robust and efficient approach for vessel analysis in prostate biopsy samples with a standardized workflow.

The proposed pipeline can be extended for analysis of blood vessel distribution in diverse cancer types. Given the distinctive variations in BV patterns across various tissues and cancer categories, it is anticipated that the proposed segmentation model may necessitate re-training or fine-tuning on histopathology images of the specific pathology. A study involving adaptation of the developed technique is presented in section 2 of the Supplementary Material, where the same segmentation deep learning model is trained and tested on a separate cohort of colorectal cancer patients. In addition, the proposed deep learning model can be potentially adapted to operate as part of image analysis workflows and libraries of existing open source bioimage analysis software, such as QuPath (Bankhead et al., 2017).

Combining information from CD31, CD34 and Collagen IV staining provided BV segmentation and detection capabilities of the DL model, as compared to using just CD31 and CD34, or CD31. This result suggests that these staining techniques can provide complementary information on the presence and location of blood vessels, enhancing thus the performance of the segmentation algorithm. In addition, using multiple histopathology images is expected to render the model less prone to artifacts caused by improper sample preparation, staining artifacts, or noise introduced by the imaging equipment.

Deep learning-based methods have been previously proposed for BV detection and classification in H&E histopathology images of lung adenocarcinoma (Vu et al., 2019) (Yi et al., 2018), oral cancer (Fraz et al., 2018), gastric cancer (Noh et al., 2023), pancreatic cancer (Kiemen et al., 2022) and kidney tissue (Bevilacqua et al., 2017). Some studies have also analyzed additional markers obtained by CD34 and/or CD31 staining for BV analysis in samples of lung cancer (Yi et al., 2018) and colorectal cancer (Kather et al., 2015). In the present study, the rationale for using these particular markers is that immunostaining of CD31 (endothelial cell protein) and CD34 (vascular endothelial cell protein and neo angiogenesis) have been reported to enable accurate assessment of microvessel density in prostate cancer subjects (Miyata et al., 2015). In addition, collagen IV immunostaining which is present in vascular basement membranes can provide complementary information on identification of BV (Gross et al., 2021). Ongoing efforts include training the presented model using standard H&E images and comparing its performance in BV detection against the proposed three-marker model.

Due to the limited availability of images with ground truth BV annotations, patch-based processing, combined with simple data augmentation by applying image rotation by multiples of 90 ° . Appropriate data augmentation transforms were employed for training the model. We experimented with additional augmentation strategies, incorporating random contrast and affine transforms, and calculated the performance metrics in the test dataset for each model, as shown in Supplementary Table S1. However, such transforms compromised the generalization capabilities of the model. A possible explanation is that the features of vessels in microscopy images do not present high intricate variations, therefore a very simple transformation can prove more beneficial. Using more complex transformations can potentially introduce variations that do not reflect the actual distribution of the data.

The full slide segmentation masks were reconstructed by stitching the generated binary patches and averaging in the overlapping areas, which may introduce distortions and spatial variations in the quality of the final reconstructed segmentation. Obtaining additional ground truth annotations is expected to further improve the generalization capability of the model. However, manual segmentation can be time consuming and expensive. Semi-supervised learning techniques, such as co-training (Hady and Schwenker, 2008), can make use of the abundant available un-labeled images to leverage information about the data structure and distribution, therefore enhancing the training process and the model performance.

A limitation of the model performance assessment in the test dataset arises from the fact that the ground truth annotations were derived by manually drawing polygons that outlined the approximate shape of the BVs. This limitation is expected to compromise the reliability of the dice similarity coefficient calculation, which received relatively lower values, due to the absence of exact vessel delineation in the ground truth segmentation masks. However, the trained model marked excellent precision and recall, which was expected given that object detection tasks are less dependent on the exact object geometry. Ongoing efforts involve utilization of semi-automated algorithms, such as the segment anything model (SAM) (Kirillov et al., 2023), to refine the manual segmentation maps and produce accurate ground truth BV annotations.

Using the segmented blood vessels, we carried out analysis investigating how blood vessels is associated with the Gleason grade as well as biochemical recurrence. We found that higher the grade, the patient tends to have more vessels in count and area. We also found significant associations between the blood vessel count and biochemical failure/disease recurrence. In future analyses, we plan to evaluate the spatial relationships between blood vessels and surrounding cell response. In addition, identifying different types of blood vessels, such as capillaries, veins and arteries can potentially provide valuable information in prostate cancer progression. A future step of this study would involve incorporating additional biomarkers in the proposed pipeline to detect different types of blood vessels and correlate each one of them with the Gleason score.

Another matter warranting further exploration is potential fragmentation of tissue resulting from the sectioning plane. This can result in vessels appearing as disconnected structures in microscopy images, which may be counted as separate BVs, overestimating thus the BV density. To mitigate this limitation, morphological closing was applied on the generated segmentation masks, which is expected to connect fragmented BV components that are in proximity. However, in cases where the fragments are highly disjointed, the accuracy of BV count may be compromised. Ongoing efforts would involve automated detection of structures that may have been affected by fragmentation to correct for any potential bias.

In this study, the U-net architecture was employed, which is a well-established DL architecture for medical image segmentation. More recently, DL models including vision transformers (ViT) (Dosovitskiy et al., 2021), denoising diffusion probabilistic models (DDPM) (Ho et al., 2020), or zero-shot approaches such as the segment anything model (SAM) (Kirillov et al., 2023) have demonstrated excellent performance in semantic segmentation (Wu et al., 2023), (Ranftl et al., 2021) and object detection (Li et al., 2022), (Chen et al., 2022) tasks. Ongoing efforts would involve training multiple models for the given segmentation task, to determine the optimal deep learning architecture.

## 5 Conclusion

In conclusion, a pipeline was introduced for automated detection and analysis of blood vessel distribution in histopathology images of the prostate. A deep learning model was trained using various combinations of immunostaining images, demonstrating excellent capability to accurately identify blood vessels in prostate biopsy samples. Furthermore, the trained model was utilized to derive features based on vessel size and distribution in a larger cohort of prostate cancer patients, which were in turn analyzed with respect to the disease progression. The presented methodology is expected to improve the efficiency and standardization of biopsy sample analysis, potentially leading to better understanding of the pathophysiology, diagnosis and staging of prostate cancer.

## Data Availability

The raw data supporting the conclusion of this article will be made available by the authors upon request.
