# Supplementary material for: Deep learning-based automated pipeline for blood vessel detection and distribution analysis in multiplexed prostate cancer images
Source: Front Bioinform. 2024 Jan 23;3:1296667. doi: 10.3389/fbinf.2023.1296667 (PMC10844485; doi:10.3389/fbinf.2023.1296667)
Supplement: Supplementary file 1 [file DataSheet1.pdf]

frontiers

Supplementary Material

Deep learning-based automated pipeline for blood vessel detection and distribution analysis in multiplexed prostate cancer images.

Grigorios M. Karageorgos[1], Sanghee Cho[1], Elizabeth McDonough[1], Chrystal Chadwick[1], Soumya Ghose[1], Jonathan Owens[1], Kyeong Joo Jung[2], Raghu Machiraju[2], Jung, Robert West[3], James D. Brooks[4], Parag Mallick[5], and Fiona Ginty[1]

[1]GE Research, Niskayuna, NY, USA.
[2]Department of Computer Science and Engineering, The Ohio State University, Columbus, OH, USA.
[3]Department of Pathology, Stanford University School of Medicine, Stanford, CA, USA.
[4]Department of Urology, Stanford University School of Medicine, Stanford, CA, USA.
[5]Canary Center for Cancer Early Detection, Department of Radiology, Stanford University School of Medicine, Stanford, CA, USA.

*** Correspondence:**
Grigorios M. Karageorgos
grigoriosmarios.karageorgos@gehealthcare.com

1) Model performance assessment for different data augmentation strategies.

TABLE – S1: Segmentation performance metrics of the RGB model in the test dataset for three different data augmentation strategies during training: 1) Simple 90° rotation, 2) 90° rotation + Contrast, 3) 90° rotation + Affine transforms

		Annotator 1						Annotator 2					
		**S1**	**S2**	**S3**	**S4**	**S5**	**S6**	**S1**	**S2**	**S3**	**S4**	**S5**	**S6**
**90° rotation**	**DSC**	0.70	0.68	0.79	0.61	0.68	0.80	0.72	0.61	0.79	0.74	0.60	0.75
	**Precision**	0.95	0.91	0.98	0.91	0.86	0.98	0.95	0.91	0.97	1.00	1.00	0.88
	**Recall**	0.98	0.94	0.98	0.97	1.00	0.98	0.96	0.83	1.00	0.97	0.88	1.00
	**FP**	3	3	1	3	3	1	3	3	2	0	0	5
	**FN**	1	2	1	1	0	1	2	6	0	1	3	0
**90° rotation + Contrast**	**DSC**	0.66	0.62	0.78	0.49	0.63	0.80	0.66	0.55	0.76	0.72	0.56	0.76
	**Precision**	0.96	0.69	0.87	0.86	0.88	0.98	0.93	0.69	0.84	0.94	0.96	0.91
	**Recall**	1.00	0.83	0.99	0.97	1.00	0.98	0.96	0.74	1.00	0.97	0.92	1.00
	**FP**	3	11	10	5	3	1	5	11	12	2	1	4
	**FN**	0	5	1	1	0	1	3	9	0	1	2	0
**90° rotation + Affine**	**DSC**	0.63	0.59	0.71	0.42	0.69	0.74	0.59	0.51	0.77	0.65	0.59	0.63
	**Precision**	0.91	0.93	0.94	0.84	0.90	0.93	0.89	0.93	0.94	0.89	0.90	0.84
	**Recall**	0.97	0.84	0.94	0.97	0.95	0.95	0.94	0.75	0.97	0.92	0.82	0.97
	**FP**	6	2	4	6	2	3	7	2	4	4	2	7
	**FN**	2	5	4	1	1	2	4	9	2	3	4	1

2) Application of automated blood vessel segmentation in colorectal cancer images.

In this section, the feasibility of the developed pipeline to detect blood vessels in multiplexed colorectal cancer images is demonstrated. The information presented in this section relies on data previously published in a study [1]. N=11 TMAs from formalin fixed paraffin-embedded (FFPE) tissue blocks were prepared from 388 patients with stage II and III colorectal cancer (n = 287 stage III patients). The punches were taken from the center of the tumor based on identification by a Pathologist (J.S., Memorial Sloan Kettering Cancer Center). All Centers provided ethical approval for this study and informed consent was obtained from all participants (NIB12-0034). This was a retrospective study, and the patients were recruited during 2005–2012. The TMAs underwent multiplexed immunofluorescence imaging similarly as described in section 2.2 of the main manuscript. N=32 cores corresponding to different subjects were randomly selected and were manually annotated for blood vessels by an expert biologist, as described in section 2.3 of the main manuscript. Independent annotated patient core images (N=27) were selected to form the training/validation dataset, while the remaining images (N=5) were held out to test the generalization capability of the model. Data pre-processing, model training and testing was carried out using the same methodologies as in sections 2.3, 2.4 and 2.5 of the revised manuscript. The RGB model was used, combining CD31, CD34 and Collagen IV images. **Figure S1 A-C,** illustrates three example CD31 images corresponding to different subjects in the test dataset. The ground truth annotations are overlaid in white outlines. The green, cyan, and magenta outlines illustrate the true positive, false positive and false negative segmentations masks that were generated by the RGB model. The average dice similarity, precision and recall were 0.64 (SD 0.14), 1.00 (SD 0.00) and 0.97 (SD 0.05), respectively, demonstrating the feasibility of the proposed pipeline to accurately detect BVs in multiplexed images of colorectal cancer.

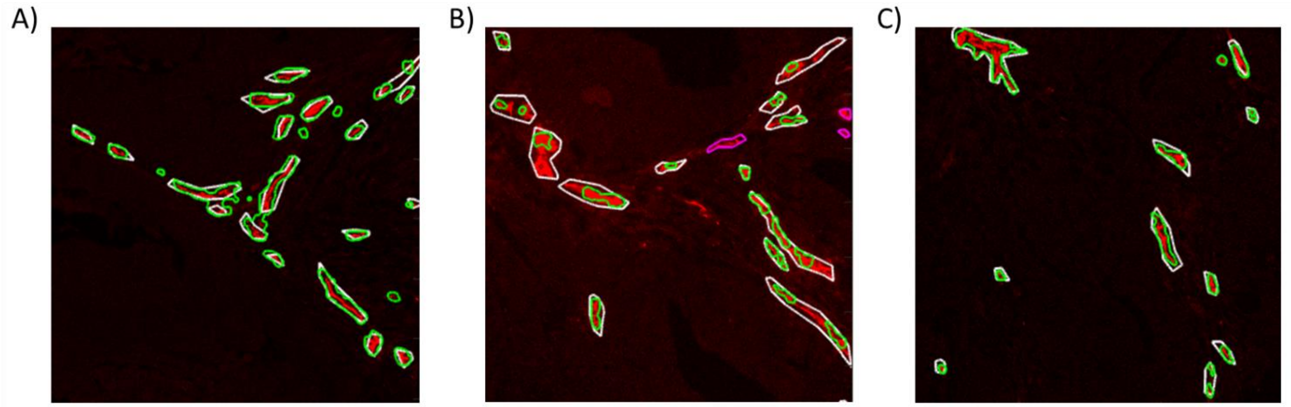

Figure S1. A-C) Three example CD31 images corresponding to three patients with colorectal cancer in the test dataset. The ground truth annotations are overlaid in white outlines. The green, cyan and magenta outlines illustrate the true positive, false positive and false negative segmentations masks that were generated by the RGB segmentation model.
